# Supratherapeutic International Normalized Ratio in a Patient on Warfarin Following Initiation of Brexpiprazole Pharmacotherapy: A Case Report

**DOI:** 10.7759/cureus.70457

**Published:** 2024-09-29

**Authors:** Joby H Elias, Leo W Olney, Paul M Frederick, Megumi Yokote, Jagadeesh Herur

**Affiliations:** 1 Clinical Pharmacy, Western Health, Melbourne, AUS; 2 Psychiatry, Western Health, Melbourne, AUS; 3 Geriatric Psychiatry, Western Health, Melbourne, AUS

**Keywords:** brexpiprazole, drug-drug interaction, gastrointestinal bleed, geriatric psychiatry, pharmacokinetics, psychiatry, warfarin toxicity

## Abstract

Many medications have established interactions with warfarin, potentially affecting international normalized ratio (INR) levels and increasing the risk of bleeding complications. We present the case of a 74-year-old female inpatient with late-onset schizophrenia who was initiated on brexpiprazole while concurrently receiving warfarin therapy for a ventricular thrombus. Despite INR levels being within a range of 1.8 to 2.7 prior to brexpiprazole initiation, the patient's INR increased sharply to 6 within five days, leading to a potential gastrointestinal bleed that required medical admission. While the manufacturer’s in-vitro studies suggest that brexpiprazole does not displace warfarin from albumin, both drugs exhibit greater than 99% protein binding. This case highlights the critical need for close monitoring of INR levels when initiating brexpiprazole in patients on warfarin therapy, due to the potential for warfarin displacement from plasma proteins and subsequent bleeding complications. This interaction warrants further exploration, and clinicians should exercise caution when using these medications concomitantly.

## Introduction

Antipsychotic medications are commonly prescribed for various psychiatric conditions, and their interactions with other drugs are well-documented in clinical practice. Brexpiprazole, a relatively newer antipsychotic, has demonstrated efficacy in treating schizophrenia and affective disorders [1]. Despite manufacturer in-vitro studies suggesting no interaction with warfarin [1], our clinical observation highlights an instance where the coadministration of brexpiprazole and warfarin was associated with a marked elevation in international normalized ratio (INR) levels-a measure of blood clotting time used to monitor warfarin therapy. This increase in INR likely contributed to a gastrointestinal bleed in our patient.

Warfarin, a vitamin K antagonist, is widely used in the prevention of thromboembolic events but requires careful monitoring due to its narrow therapeutic index and extensive list of drug-drug interactions [2]. Many medications have been implicated in altering the pharmacokinetics of warfarin, leading to fluctuations in INR levels and potentially serious complications [2]. One established interaction involves the displacement of warfarin from serum albumin by drugs with a high binding affinity for plasma proteins [3].

This case report underscores the clinical implications of the brexpiprazole-warfarin interaction, emphasizing the need for close INR monitoring and careful consideration when prescribing brexpiprazole in such cases. Additionally, it prompts further investigation into the underlying mechanisms of interaction between these medications and potential strategies to mitigate patient risk.

## Case presentation

The patient is a 74-year-old female who presented to the hospital with a history of chest pain, dry cough, and shortness of breath. Her symptoms had progressively worsened over four days, leading to her arrival at the emergency department via ambulance. Her medical history was notable for intermittent leg swelling over the past year, and she was unable to lie flat due to breathlessness. Her family also reported a long-standing history of psychotic symptoms, which began following the death of a loved one two decades ago and had worsened over the last 10 years. Additionally, she had a history of heavy alcohol use, drinking three large bottles of whiskey or vodka weekly. Her medical history included type 2 diabetes mellitus and hypertension, and her medications consisted of metformin 1000 mg once daily and perindopril 5 mg once daily.

Laboratory tests showed that her transaminases and albumin levels were within the normal range, and her estimated glomerular filtration rate (eGFR) was above 90 (Table 1). An electrocardiogram revealed ST changes in the inferior leads, and she was diagnosed with an inferior STEMI. Cardiac catheterization revealed 95% stenosis in the mid-left anterior descending artery (LAD) and apical ballooning syndrome (ABS). Additionally, a transthoracic echocardiogram (TTE) revealed a dilated left ventricle and a left ventricular thrombus, leading to the initiation of warfarin sodium 3 mg daily, targeting an INR between 2 and 3. Alongside her existing medications, the patient was started on bisoprolol 1.25 mg once daily, ticagrelor 90 mg twice daily, dapagliflozin 10 mg once daily, and rosuvastatin 40 mg once daily. After these treatment changes, she was transferred to the medical ward. During her stay, her cardiac function and symptoms, including chest pain and shortness of breath, stabilized, and her INR remained within the range of 1.2 to 2.7.

**Table 1:** Patient Laboratory Values at Significant Stages of Admission

Test type	Value range	On the day of the presentation	Day of warfarin initiation	Admission to psychiatric ward	24 hours after brexpiprazole initiation	Brexpiprazole cessation	48 hours after brexpiprazole cessation
INR	0.8–1.1 or 2–3 on warfarin	1.1	1.2	1.8	5.5	6.0	2.2
Prothrombin time (seconds)	11–13.5	12.8	13.0	–	54.8	59.6	24.0
Sodium (mmol/L)	136–147	141	141	139	143	–	144
Potassium (mmol/L)	3.5–5.3	4.4	3.9	4.1	3.9	–	3.7
Chloride (mmol/L)	96–112	107	97	97	103	–	107
Bicarbonate (mmol/L)	19–29	23	33	25	27	–	23
Blood urea (mmol/L)	1.5–7.2	6.3	7.3	7.3	18.2	–	19.1
Creatinine (µmol/L)	50–120	56	–	–	–	–	–
Blood glucose (mmol/L)	3.9–5.6	6.3	5.5	–	5.1	–	–
Troponin (ng/L)	<14	391	316	–	–	–	–
Albumin (g/dL)	30–54	34	35	31	36	–	–
eGFR (mL/min/1.73m^2^)	>90	>90	78	>90	>90	–	–
WBC (k/μL)	3.8–10.5	7.9	9.6	9.1	–	–	–
Neutrophils (1.5–8.0 × 10^9^/L)	1.5–8.0	6.0	7.1	5.6	–	–	–
RBC (million/mm^3^)	4.3–5.9	3.9	4.0	3.7	3.1	–	3.2
Hemoglobin (g/L)	115–185	108	116	107	69	71	78
Hematocrit (%)	39–50%	32	36	36	30	31	31
Platelet count (K/μL)	150–400	290	308	293	276	280	272
CRP (mg/L)	<5	30	–	–	–	–	–

One week after admission and the initiation of warfarin, following medical stabilization with an INR of 1.8, the patient was transferred to an older age psychiatry unit. Psychiatric consultations, including clinical interviews and mental state examinations, concluded that the patient had a potential diagnosis of late-onset schizophrenia. Brexpiprazole 1 mg once daily was initiated to manage her psychotic symptoms, as it was chosen for its minimal cardiovascular risk profile and efficacy in treating psychosis [4].

Despite an improvement in her psychotic symptoms, the patient began to show signs of medical deterioration approximately 24 hours after brexpiprazole initiation. Her systolic blood pressure dropped to 80 mmHg, and she developed shortness of breath and abdominal pain. During her 72-hour stay in the psychiatric unit, her hemoglobin, initially 107 g/L on admission, decreased to 69 g/L, while her red cell count (RCC) dropped from 3.77 to 3.13, and her INR increased to 5.5. Consequently, physicians reduced her warfarin dose from 3 mg to 1 mg, with daily monitoring of INR.

Forty-eight hours after the warfarin dose reduction, the patient was transferred back to the medical ward, where she received a blood transfusion and underwent close monitoring and investigations for potential gastrointestinal hemorrhage. Despite the reduction in warfarin dosing, the patient’s INR remained elevated at 5.5. The treating psychiatrist discontinued brexpiprazole approximately four days after its initiation, as it was the only newly added medication coinciding with the patient’s rise in INR. Despite stopping brexpiprazole, the patient’s INR increased further to 6 within the following 24 hours. Approximately 48 hours after discontinuing brexpiprazole and five days after reducing the warfarin dose, the patient’s INR began to decrease, eventually reaching 2.2. The decision was then made to replace warfarin with therapeutic enoxaparin, with plans to initiate a new antipsychotic once the patient was medically stable.

## Discussion

This clinical case highlights a potential drug-drug interaction, with the key concern being the possible displacement of warfarin by brexpiprazole from serum albumin. This displacement could have led to an increased INR and subsequent medical deterioration.

Warfarin is an anticoagulant that works by inhibiting the synthesis of vitamin K-dependent clotting factors. It is an acidic compound that is highly protein-bound, with approximately 99% of the drug bound under normal physiological conditions [5]. This high degree of protein binding means that only a small fraction of warfarin exists in its free, active form. Any factor that displaces warfarin from albumin can significantly increase the free fraction, thereby enhancing its anticoagulant effect, potentially leading to elevated INR levels and an increased risk of bleeding [6].

Brexpiprazole, an atypical antipsychotic, is used for the treatment of psychosis and is beneficial in managing affective symptoms [7]. It has a high affinity for a variety of serotonergic, dopaminergic, and noradrenergic receptor subtypes, notably acting as a partial agonist at 5-HT1A and D2 receptors and an antagonist at 5-HT2A receptors, which contributes to its efficacy [7]. Brexpiprazole is also highly protein-bound in plasma (greater than 99%) to serum albumin and α1-acid glycoprotein [1], similar to warfarin. Therefore, there is potential for it to displace other highly protein-bound drugs from albumin. While in-vitro studies referenced in the product monograph suggest that brexpiprazole does not significantly alter the protein binding of warfarin [1], the clinical relevance of these findings in elderly patients with complex medical conditions warrants careful consideration.

In elderly patients, several physiological changes affect drug pharmacokinetics and pharmacodynamics. Altered body fat composition, decreased renal and hepatic function, and polypharmacy all increase the risk of drug-drug interactions. These factors can lead to higher free drug levels and increased sensitivity to medications [8]. In this case, the patient’s age and substantial medical history may have made her particularly susceptible to the hypothesized interaction.

The patient’s marked INR increase and subsequent medical deterioration following the initiation of brexpiprazole suggest a potential pharmacokinetic interaction. The rapid INR rise from 1.8 to 5.5 could indicate a significant increase in warfarin levels. Additionally, the patient’s INR continued to rise from 5.5 to 6.0 despite the cessation of brexpiprazole 48 hours earlier, which may be due to brexpiprazole's extensive half-life of 91.4 hours as listed by the manufacturer [1]. The sequence of events supports this hypothesis. Despite reducing the warfarin dose from 3 mg to 1 mg, the INR continued to increase for approximately five days, suggesting an ongoing pharmacokinetic interaction. After discontinuing brexpiprazole, the patient’s INR gradually decreased to 2.2 within 48 hours, further supporting the idea that brexpiprazole may have contributed to the elevated INR.

While no other published case reports directly link brexpiprazole to changes in INR levels, a case report by Tuman in 2017 described a similar situation in which the initiation of aripiprazole was associated with an increase in INR in a patient undergoing warfarin therapy [9]. In this case, a 32-year-old female with unspecified schizophrenia spectrum disorder had an initial INR of 2.18 while on warfarin for thrombosis prophylaxis. After one week of aripiprazole treatment, her INR sharply increased to 8. Following the discontinuation of aripiprazole, her INR decreased to 2.24 within a week [9]. The authors speculated that the rise in INR could be due to competitive protein binding, given that both aripiprazole and warfarin have a high affinity for serum albumin [9]. This potential for a similar interaction with brexpiprazole underscores the importance of vigilant INR monitoring in patients receiving both medications.

The Adverse Drug Reaction Probability Scale, or Naranjo Scale, developed by Naranjo and colleagues in 1991, helps standardize the assessment of causality in adverse drug reactions [10]. Applying this scale to the current case suggests a possible adverse drug reaction. The rapid INR increase following brexpiprazole initiation, followed by a decrease after its discontinuation, points toward an adverse reaction. Considering the Naranjo scale’s criteria, including the timing of the reaction (+2) and improvement upon drug discontinuation (+1), and accounting for the potential influence of the warfarin dose reduction as an alternative cause (-1), the total score would be 2. This score indicates a "possible" adverse drug reaction [10], emphasizing the need for careful monitoring when these drugs are co-administered, particularly in elderly patients.

Several limitations of this case must be considered. The observational nature of this report precludes establishing a definitive cause-and-effect relationship between brexpiprazole and increased INR. It is important to acknowledge that warfarin dose reductions may have a delayed effect on INR, as changes in dosage can take two to three days to be reflected [11]. Thus, the observed decrease in INR could result from a combination of factors, including the cessation of brexpiprazole and the predictable delayed effect of warfarin dose adjustments. However, given the timeline of events, it is unlikely that this lag effect alone accounts for the INR reduction. Other factors, such as the patient’s concomitant medications, comorbidities, and potential dietary variations, could have influenced warfarin metabolism or pharmacodynamics, contributing to increased INR levels. Additionally, the absence of serial measurements of free warfarin levels limits the ability to conclusively determine an increase in serum concentrations of warfarin.

## Conclusions

This case highlights the potential for brexpiprazole to displace warfarin from albumin, leading to elevated INR levels. The rapid increase in INR observed after the initiation of brexpiprazole, despite adjustments to warfarin dosing, underscores the significance of this interaction. Given the high protein-binding properties of both drugs, healthcare providers should be vigilant when prescribing them concurrently, especially in elderly patients who often have multiple comorbidities and altered pharmacokinetics. Regular monitoring of INR levels is essential to prevent adverse effects and ensure patient safety.

Further studies are needed to explore the interaction between brexpiprazole and warfarin. Future research could focus on understanding the mechanisms behind these interactions, the influence of patient-specific factors such as age and comorbidities, and the development of guidelines for the safe co-administration of these medications. Given the ethical concerns that randomized controlled trials may pose in this context, case-based evidence like this report plays a crucial role in informing clinical practice regarding such interactions.
